# Trichloroethylene: Johnson et al.’s Response

**Published:** 2004-08

**Authors:** Paula D. Johnson, Brenda V. Dawson, Stanley J. Goldberg, Mary Z. Mays

**Affiliations:** University Animal Care, The University of Arizona, Tucson, Arizona, E-mail: pauladj@email.arizona.edu; University of Auckland, Faculty of Medical and Health Sciences, Auckland, New Zealand; Congenital Cardiology, University Physicians, Tucson, Arizona; Department of Family and Community Medicine, University of Arizona Health Sciences Center, Tucson, Arizona

We share Hardin et al.’s belief that any apparent conflict of interest should be reported. We note that Brent provided testimony for the defense in TCE litigation, notably for the same case in which Goldberg (based on his extensive epidemiologic and laboratory research on the effects of TCE) acted as an expert witness for the plaintiff. We did not report Goldberg’s experience acting as an expert witness because the point of expert witness is to provide unbiased, factual explanations of extant data. We believe this does not constitute a conflict of interest; we have included a caveat about extrapolating data to humans in our publications. To our knowledge none of our data have been used inappropriately.

The work published in 1993 (Dawson et al.) and in 2003 (Johnson et al.) was actually performed during a much shorter period of time. Many extraneous factors contributed to the late publication of the 2003 paper. Data from our previous work was included in the more recent paper because we needed “boundary values” between or below which we were looking for a threshold or a critical level. This was a long-term study, and it would have been an inappropriate use of animals to repeat the earlier animal studies for those groups. We should have stated more clearly that we were using the groups already studied to prevent repetition and to conserve animal resources, as recommended by the Animal Welfare Act (1990); however, we did refer to our previous paper. Our 2003 publication contained new data as well as previously published data. We welcome this opportunity to clarify our method.

Our alleged reclassification of defects in our Table 2 (Johnson et al. 2003) merely reflects careful reevaluation by the cardiologist and minor updates in terminology that mirror current clinical usage to clarify the nature of a defect (e.g., great vessel defect vs. the more specific aortic hypoplasia; L-transposition vs. abnormal looping, etc.). There are other minor numerical differences in the tables (Table 2, Johnson et al. 2003, and Tables 1 and 3, Dawson et al. 1993), not remarked upon by Hardin et al., which derive from the more extensive statistical analysis in the later paper. In an apparent typographic error, we failed to report a pulmonary valve defect for the 1.5 ppm TCE in the 2003 paper. This should have been included in Table 2; however, it would not have changed the number of hearts with defects.

Again, because this was a long-term continuous project, we did use all of the controls together in a cumulative manner. We used the larger sample size with data collected over a long period because it increases the generalizeability of our data, demonstrating clearly the background rate and the variability around rate estimates. Control values were consistent throughout our studies. The larger sample size did increase statistical power somewhat in our most recent paper (Johnson et al. 2003), again without inappropriate use of further valuable animal resources. It should be noted that the increase in statistical power is small compared to the increase generated by the effect sizes and the increase in the number of dose groups—data that can only be generated in a long-term project.

Our statistical analysis was simple and conventional. Hardin et al. are incorrect in stating that the differences at the 1.5-ppm dose were statistically significant in our recent paper (Johnson et al. 2003). The *p*-values were reported in Figures 1 and 2 of our paper as 0.14 and 0.08, respectively, values not conventionally seen as statistically significant. Different levels of statistical significance used in each of the studies for each of the groups were carefully listed in the tables and figures and explained in the text.

There are many references in the scientific literature about effects of halogenated hydrocarbons on development. We included only a few of these in our articles. We are a multidisciplinary team and have studied both TCE and its major metabolites, often basing some of our work on the findings of others in the field without duplicating the work of others. We have consulted with other prominent researchers in the field from time to time in establishing our experimental design or in interpreting our results. We have found only heart defects associated with these compounds, despite looking for other effects. This work has been consistent with the original epidemiological studies on which our laboratory work was based. We have been funded by government and other nonbiased agencies requiring competitive grant application and accountability. We have presented our results as peer-reviewed published articles in excellent journals. Our work has all been carried out at The University of Arizona. A major strength of our studies was microdissection of each heart by investigators fully versed in the pathology of congenital cardiac malformations as well as noncardiac anatomy.

We fully agree with Hardin et al. that studies in this area “have potential for important health and public policy implications, so it is particularly important for the scientific and regulatory communities to have confidence in the conduct and reporting of those studies.” We believe that our studies have been rationally planned, are statistically and scientifically sound, and are of value for this purpose. We welcome this opportunity for postpublication discussion of results.
